# Construction and evaluation of a large radiation detector for Positron Emission Tomography applications

**DOI:** 10.1088/1748-0221/19/04/p04034

**Published:** 2024-04-26

**Authors:** R.R. Raylman, A.V. Stolin, G. Jaliparthi, P.F. Martone

**Affiliations:** Center for Advanced Imaging, Department of Radiology, West Virginia University, 64 Medical Center Dr, Morgantown, WV, U.S.A.

**Keywords:** Gamma camera, SPECT, PET PET/CT, coronary CT angiography (CTA), Detector design and construction technologies and materials, Detector cooling and thermo-stabilization

## Abstract

Large arrays of pixelated scintillator potentially have application in economical construction of PET scanners. In this investigation, we constructed and evaluated a detector with an active area of 32.26 × 13.47 cm^2^. It is based on a 218 × 91 array of 1.4 × 1.4 × 15 mm^3^ LYSO elements (pitch 1.48 mm). Scintillation light is detected with a 5 × 12 array of silicon photomultipliers (SiPM) arrays. Each array consists of an 8 × 8 array of 3 × 3 mm^2^ (pitch 3.35 mm) SiPMs. Performance of these devices are enhanced and stabilized by cooling them. Testing revealed that the detector was able to detect 90% of the theoretically detectable 511 keV photons. The resolvability index (a measure of the ability to identify individual detector elements from background) is 0.24 ± 0.04. Additionally, the average energy resolution for the complete detector is 18.3%. These results compare well with those reported for much smaller detector modules.

## 1 Introduction

Early positron-emission scanners were based on the detection of annihilation photons first with discrete detectors [1, 2] then planar arrays of detectors [3]. Building upon this early work, contemporary PET scanners consist of rings of rectangular detector modules [4–19]. Perhaps the most important downside to this design is the necessity to utilize large amounts of scintillator and photo-sensitive devices resulting in high capital costs, especially as the price of the new generation of scintillators (LSO and LYSO), continue to increase.

In response to the high cost of complete ring PET scanners, rotating parallel detector-based systems were re-discovered [20, 21]. In addition to reduced cost and complexity, this geometry can be used to create dedicated scanners [22–25]. Furthermore, the open nature of the rotating parallel plate geometry also facilitates efficient combination with the components of a CT scanner [26]. The sizes of the detectors used to create these systems are relatively small (~ 10× ~ 10 cm^2^), limiting their application to imaging of small regions, such as the breast. Our goal is to create a large panel PET detector suitable for use of in dedicated, rotating systems intended to image larger regions, such as the head or neck [27], or as a module for use in large cylindrical PET scanners. Challenges in construction of a large panel radiation detector include maintaining good identification of detector elements, energy resolution, detection efficiency and response uniformity. This work describes the design, construction, optimization, and initial testing of this device.

## 2 Methods

PET detectors must be able to estimate positions of annihilation photon interaction points efficiently and accurately. The fundamental step in creating a radiation detector is combining a scintillator (LYSO, in this case) with the light detection devices (SiPM arrays). This combination creates the raw data (light intensities) from which event positions are estimated. Detection of scintillation light, however, is only the first step in this procedure. The analog signals produced by the detector electronics must be digitized, stored and processed. The methods to accomplish these tasks are relatively well established. The goal of this project is adaptation of these techniques to the challenges presented by construction of a large area pixelated detector.

### 2.1 Detector construction

**Figure 1.**
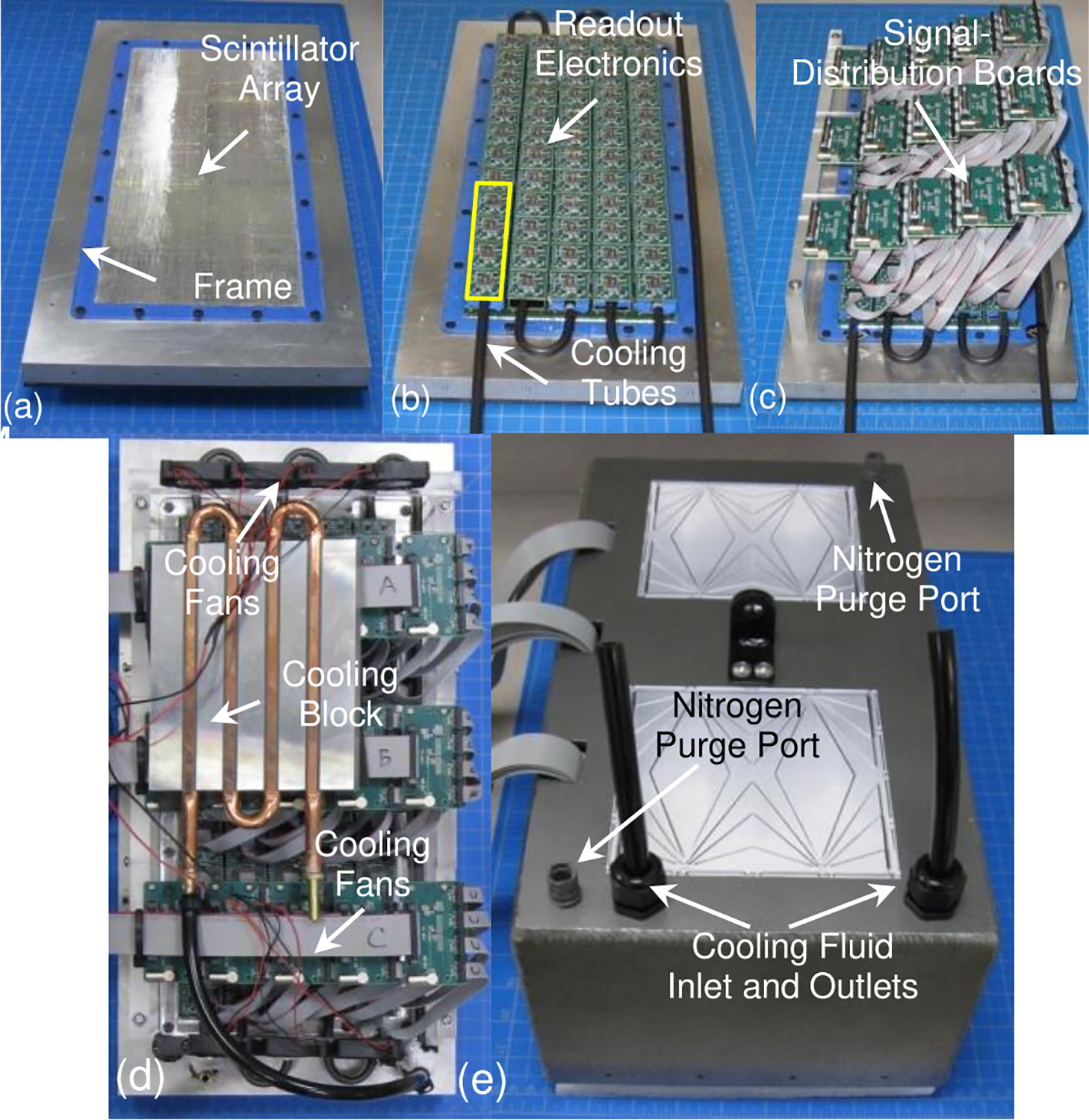
The large area PET detector. (a) Scintillator array and light guide mounted in the aluminum frame, (b) scintillator array with the SiPM readouts and cooling tubes (the yellow box shows an SiPM array group), (c) addition of the SiPM-signal distribution boards to the detector, (d) detector with full cooling system and (e) complete detector head with back cover.

The detector consists of a 218×91 array of 1.4×1.4×15 mm^3^ LYSO elements (pitch 1.48 mm) (Sichuan Tianle Photonics, Chengdu, China). The size of the scintillator array is thus 32.26×13.47×1.5 cm^3^. It was mounted in a frame (42.77 × 21.44 cm^2^) constructed from a single piece of aluminum. An acrylic light guide (3.18mm thick) was coupled to the surface of the scintillator array using an optically clear compound (Sylgard silicone elastomer (Dow Chemical, Midland, MI)). The light guide/scintillator array combination was mounted to the frame using a series of fasteners (figure 1(a)). A 5 × 12 array of silicon photomultipliers (SiPM) arrays (PA3325-WB-0808, KETEK, Munich Germany) were mounted on the light guide to detect scintillation light. These devices consist of an 8 × 8 array of 3 × 3 mm^2^ (pitch 3.35 mm) SiPMs. Each array has 0.16mm thick optical inactive area at its periphery. The arrays were connected to readout electronics (AB4T-PA33WB0808, AiT Instruments, Newport News, VA) (figure 1(b)). These diode-based devices reduce the number of analog output channels from sixty-four to four (two X and two Y channels) per SiPM array [28].

The four-channel analog output from each SiPM array was routed to a signal distribution board. This device receives the signals from four arrays, combines them for transmission on a single ribbon cable to a 16-channel control/ADC module (SIPMDAQ16 (AiT Instruments, Newport News, VA)) where the signals are digitized, reducing the required number of ADC units from sixty to fifteen. It also distributes bias voltage from the control module to the four SiPM arrays connected to the board. In addition to bias voltage, current and temperature measured by sensors on each SiPM array are transmitted to a control computer via the same ribbon cable. Thus, the sixty SiPM arrays are arranged in fifteen groups of four (figure 1(c)). This scheme was implemented to reduce the total number of data acquisition and control modules necessary to obtain data, reducing cost and complexity. The disadvantage of this approach is that a single bias voltage must be applied all four SiPM arrays that constitute a group. Unfortunately, the gain of each SiPM array can vary slightly with bias voltage due to differences during manufacturing, so biasing them with the same voltage will possibly result in non-uniform outputs for the group. To address this issue, the arrays in each group of four were gain matched. The matching process required assessment of the gain for each array by determining the position of the photopeak (511 keV annihilation photons) in an energy spectrum measured at a fixed temperature (16°C) and fixed voltage (32.5 V). SiPM arrays with similar photopeak positions were grouped in sets of four. The bias voltages for these gain-matched groups were adjusted slightly so that the average photopeak position for a group was ~650 ADC channels. The bias voltages for the fifteen groups ranged from 32.42 V to 32.65 V.

Performance characteristics of SiPMs are enhanced by reducing their temperature (reduced Johnson noise and increased gain) [29]. Hence, the detector is cooled by circulating dielectric coolant (Engineer Fluids, St. Petersburg, FL) through thermoconductive plastic tubing (Fluorotherm Polymers, Parsippany, NJ) inserted between the SiPMs and the analog readout electronics board (figure 1(b)). The fluid is also routed to a cooling block inside the detector housing to enhance heat transfer. The fluid is cooled to 2°C and pumped through the tubing with a desktop minichiller (Huber U.S.A. Inc., Raleigh, NC). While the cooling fluid is at 2°C, temperatures of the SiPM arrays ranged from 15.8°C to 16.5°C, due to the heat load from the SiPMs and readout electronics when the system is operated. Cooling efficiency is increased by mounting two banks of mini-cooling fans the detector to circulate cooled air around the detector (figure 1(d)). Finally, the detector is enclosed in an air/light-tight cover (figure 1(e)). To prevent condensation forming on the cooled detector electronics, the enclosure is purged with nitrogen gas once it has been sealed. The front face of the detector head is protected by a 3 mm thick sheet of carbon fiber.

### 2.2 Data acquisition

**Figure 2.**
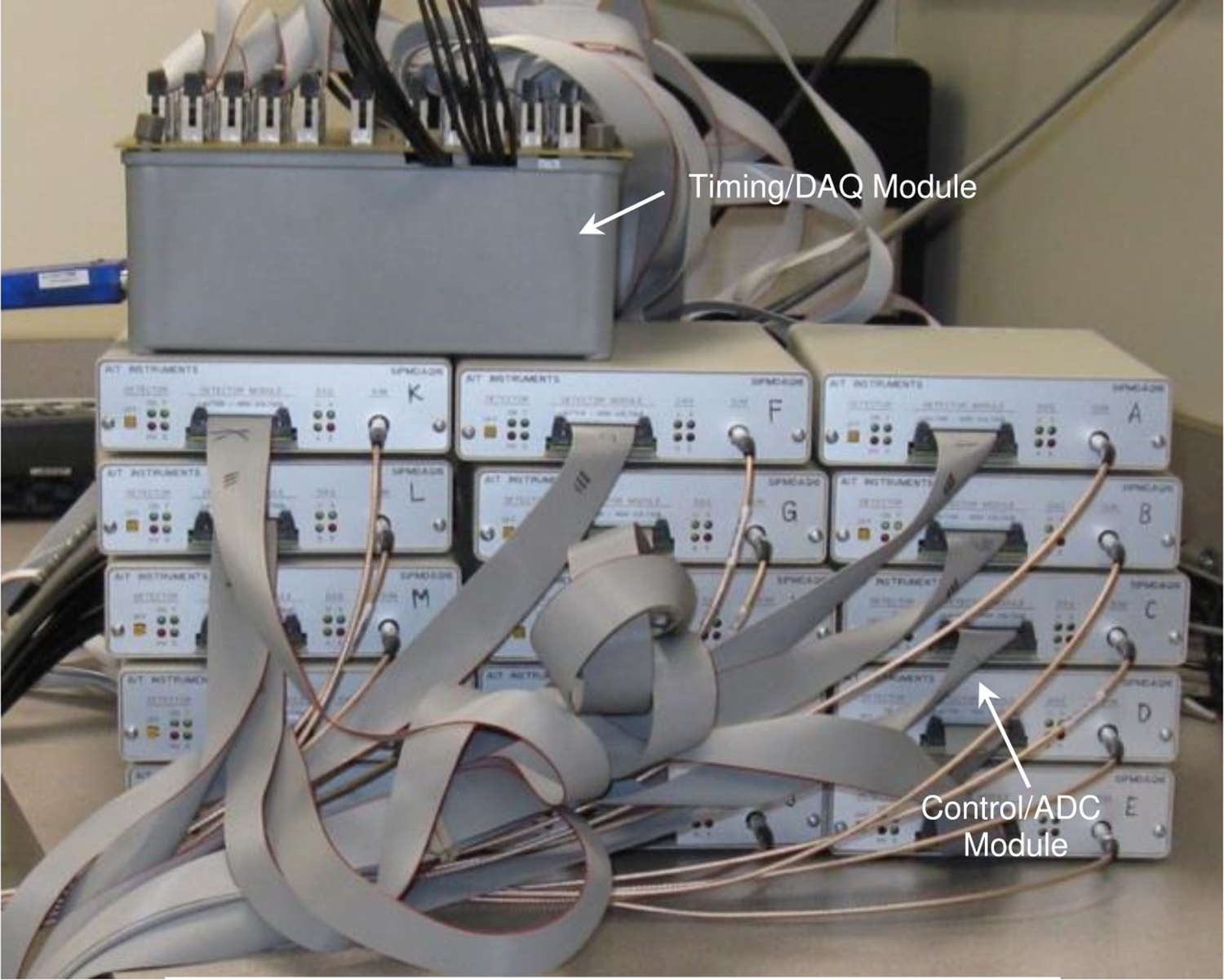
Picture showing the data acquisition system.

As noted above, the sixteen channels of analog output from the four SiPM arrays that constitute a group are digitized by a control/ADC module. Thus, fifteen 16-channel, control/ADC modules are needed to process the analog data from the detector. In addition to controlling bias voltage, and receiving SiPM array current and temperature data, these modules digitize the analog signals from the four SiPM arrays in a group using 12-bit ADCs. The modules also include circuitry to first sum and then pulse height discriminate these summed analog signals. Each digital conversion is assigned a timestamp (10 ns resolution) relative to a timing reference distributed to all fifteen modules supplied by a common 10 MHz oscillator located in a separate timing/DAQ module designed and constructed by our group. The timing reference pulses produced by the oscillator in the timing/DAQ module are distributed to the fifteen control/ADC modules. A computer user interface controls operation via USB3 communication protocols. Figure 2 shows the timing/DAQ module and the bank of fifteen control/ADC modules.

Analog-to-digital conversions are performed simultaneously in response to a TTL trigger pulse produced by a 16-channel programmable NIM-protocol-based coincidence-discriminator module (MCFD-16, Mesytec GMBH & Co. KG, Putzbrunn, Germany). This unit accepts the fifteen event energy sum signals from the control/ADC modules and the event energy sum signal from a separate detector consisting of a position-sensitive photomultiplier tube (PSPMT) (H8500, Hamamatsu, Hamamatsu City, Japan) coupled to a 5×5 cm array of 2×2×10 mm^3^ LYSO elements. The coincidence module is programmed such that each of the fifteen sum signals from the SiPM groups is in coincidence with the signal from only the PSPMT-based detector (6 ns coincidence timing window). Note that the sum signals from the large area detector were not in coincidence with each other. Digitized data, including timestamps for the conversions, are continually streamed over fifteen USB3 connections (one from each control/ADC module) to two data acquisition computers (Dell PowerEdge R340). Following completion of a data acquisition session, these data are transmitted to a processing and display computer (Dell Precision 7920) via an internal gigabit computer network.

### 2.3 Event processing

The first step in the data processing procedure is to determine which of the digitized signals belonged to the same coincidence event. To accomplish this task, the fifteen data files are scanned for entries containing near-identical timestamps (± 10 ns) and grouped into a single data entry. After a complete event is recovered, previously measured ADC pedestals are subtracted from the ADC data. These values are summed to produce a signal sum for each of the sixty SiPM arrays in the detector. The SiPM array that produced the maximum sum signal (the total signal is indicative of the amount of scintillation light collected by the array) is identified and defined as the primary SiPM array. Based on the identity of the primary array, the identities of the arrays adjacent to this array are determined, leading to the formation of a 3 × 3 matrix data set. Since the information from each SiPM arrays consists of the digitized values of four analog signals (two *X* and two *Y* channels), this 3 × 3 matrix of array signals is a set of 6 × 6 digitized analog outputs from the readout electronics (six *X* digitized readout signals × six *Y* digitized readout signals). If the signal sum from an array in this matrix does not exceed 5% of the primary array’s sum, its *X* and *Y* channels is set to zero. Next, each row (*Y*-digitized signals) and column (*X*- digitized signals) are summed to produce six *Y*-sums and six *X*-sums. These sums are summed, resulting in a total *Y* signal and a total *X* signal value. Seven percent of these values (*X* and *Y* total signals) are subtracted from each of the respective individual six *Y*-sums and six *X*-sums [30]. If a difference is less than zero, the sum is set to zero. This process reduces the signal noise at tails of the *X* and *Y* signal distributions which are mostly caused by SiPM noise and intrinsic radiation in the scintillator. The center-of-gravity (COG) of the two resulting one-dimensional digitized vectors (*X* and *Y*) are then calculated to estimate the *x*- and *y*-positions of the event’s position.

### 2.4 Detector calibration

**Figure 3.**
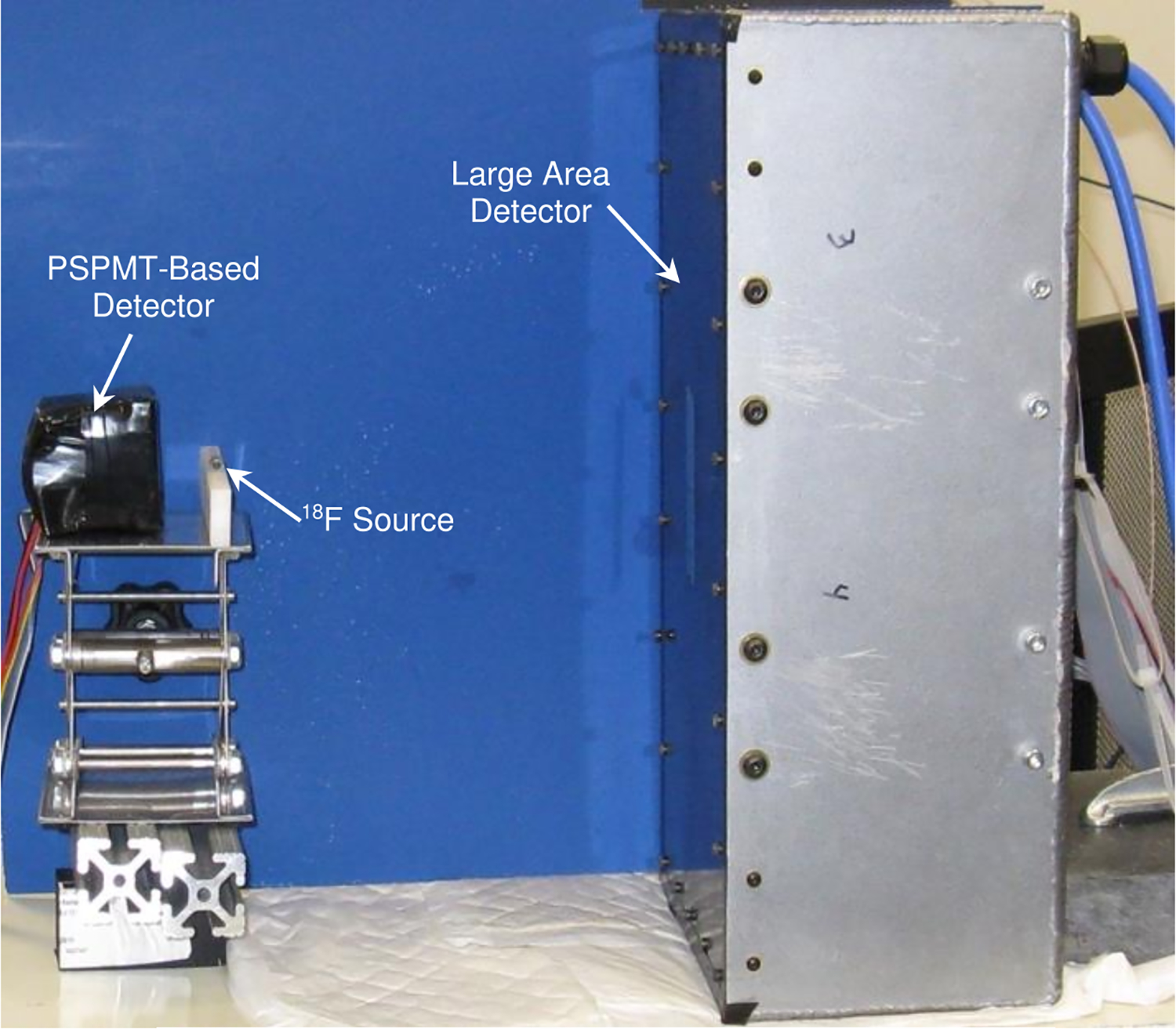
Apparatus used for all measurements.

**Figure 4.**
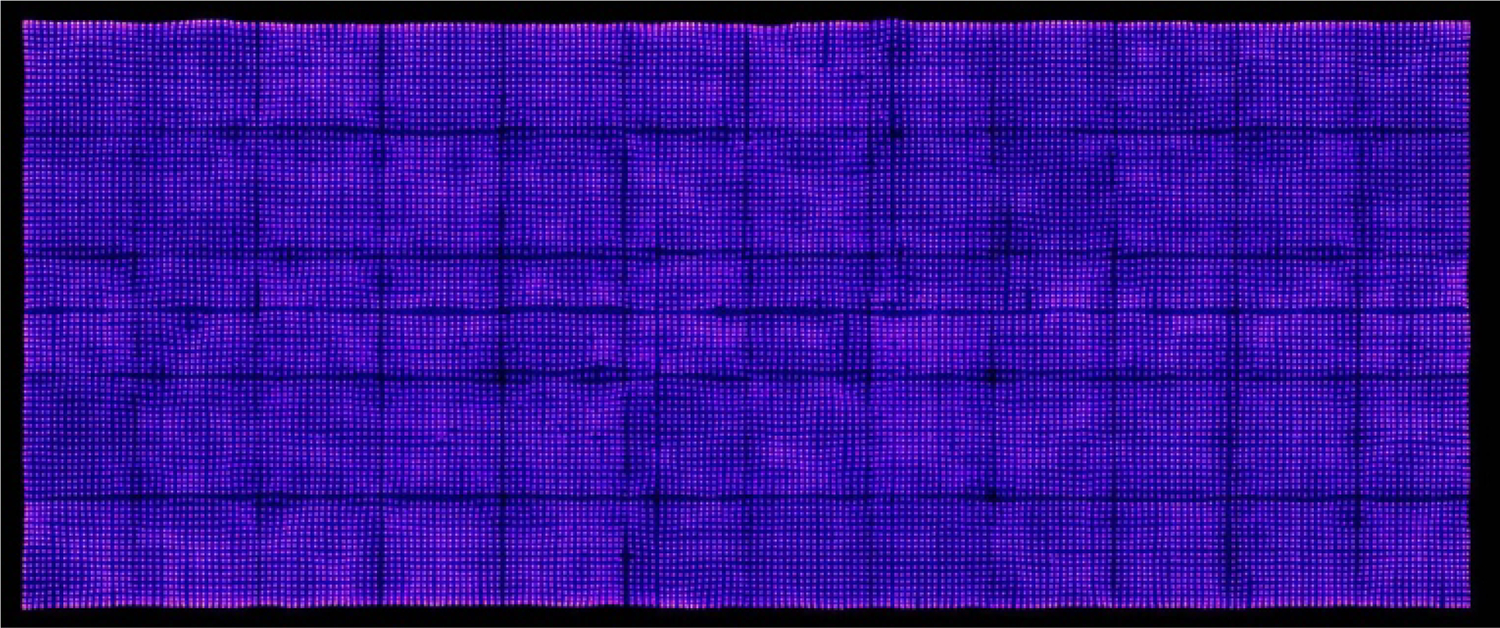
Intensity map showing positions of detector elements.

To enhance performance of the detector, several calibration steps were performed. This process required acquisition of a high statistics map of the event counts recorded. Thus, a data set consisting of approximately seventy million total coincidence events was acquired by placing a small ^18^F source between the large area detector and the PSPMT-based detector (figure 3). The COG of each event was calculated and histogrammed, with the results plotted on a digital grid representing the surface of the detector. This structure is a map of the detector element signals (figure 4). Locations of each of the 218 × 91 (19,838) detector elements were determined by performing a search for maxima in the data. These peak locations were then used to produce a set of Voronoi diagrams that were used to define regions around each peak to create a map of detector element positions (pixel map). Ideally, this map would match the regularly spaced pieces of scintillators, but due to optical effects and variations in SiPM performance, this map is not a regular grid. In addition to producing a spatial calibration map to facilitate event positioning, a method for calibrating the amount of energy deposited in the detector was implemented. Specifically, energy spectra were measured for each detector element identified in the pixel map using data from a second long data acquisition. Peak positions associated with 511 keV annihilation photon photoelectric interactions in the scintillator were determined in each spectrum. These positions were then used to create an energy calibration table relating photopeak-ADC channel number to 511 keV for each detector element.

### 2.5 Performance tests

The capabilities of the detector were assessed by testing some of the important characteristics of a PET detector. For example, its ability to accurately localize annihilation photon interactions was measured by utilizing scintillator pixel positions derived from the pixel map to calculate the resolvability index (RI) [31] for the *i*^*th*^ detector element.

$${\text{RI}}_{i}=\frac{{\text{FWHM}}_{i}}{D_{i}},$$

where FWHM_*i*_ is the average full width at half maximum (*x* and *y* directions) calculated from the fit of a two-dimensional Gaussian function to intensity distributions measured for the *i*^th^ detector element. *D*_*i*_ is the average distance between the *i*^th^ detector element and its neighboring elements measured using the pixel map described above. RI was calculated for every detector element for which the Gauss fit converged; it failed for 196 of the 19,838 total detector elements. Most of the distributions for which the fit failed were for elements located at the outer edges of the scintillator array. RI is more accurate for assessing identifiability than peak-to-valley when individual detector elements are well resolved [31]. The closer a value is to 0, the higher the resolvability of the detector element. A value of 1 indicates complete indistinguishability. RI was set to 0 for those 196 elements where the fit did not converge. Mean and standard deviation of RIs for the detector were determined from individual RI values calculated for each detector element where the Gaussian fit converged (detector elements where the fit failed were excluded from these calculations).

Energy spectra for each detector element were obtained by exposing the detector to a ^18^F point source. The location of the photopeak was identified for each spectrum. From these spectra, a global normalized detector energy was created by aligning the 511 keV photopeaks measured for each detector using the energy calibration table and summing them. Detection efficiency was calculated as the ratio of the recorded events from an ^18^F point source (detected photon energy window of 350–650 keV) to the theoretical number of detected photons. The theoretical number of detected photons was calculated by first determining the flux of annihilation photons striking each detector by accounting for strength of the source and the solid angles subtended by each detector (PSPMT-based and large detector) with the source at a known position. The theoretical maximum number of photon interactions in each detector was then calculated by multiply these fluxes by the absorption coefficients for the 15 mm and 10 mm thick pieces of pieces of LYSO used in the large area detector and the PSPMT-based detector, respectively. Detection uniformity was assessed by illuminating the detector with a ^18^F point source at approximately one and a half meters from the surface of the large area detector. The mean value and standard deviation of the events recorded in all the elements measured over the detector were calculated. The ratio between the standard deviation and the mean is reported as detection uniformity.

## 3 Results

### 3.1 Detector performance

**Figure 5.**
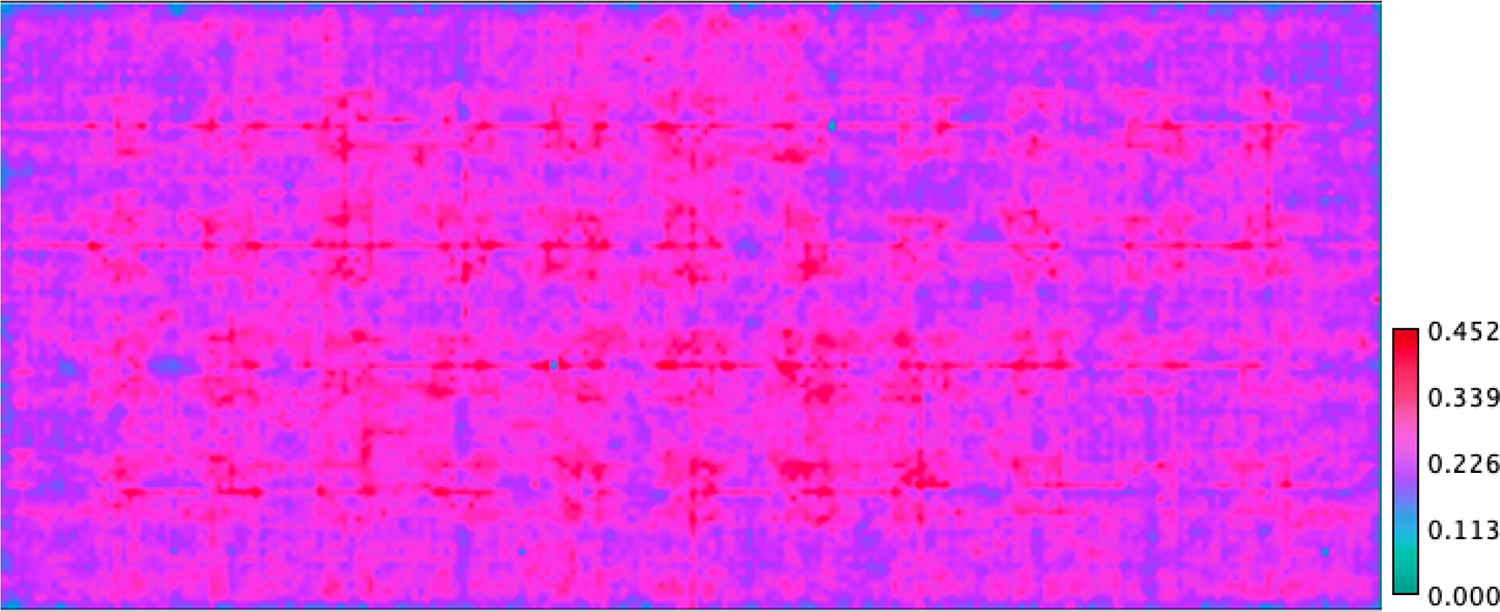
Map of RI values.

**Figure 6.**
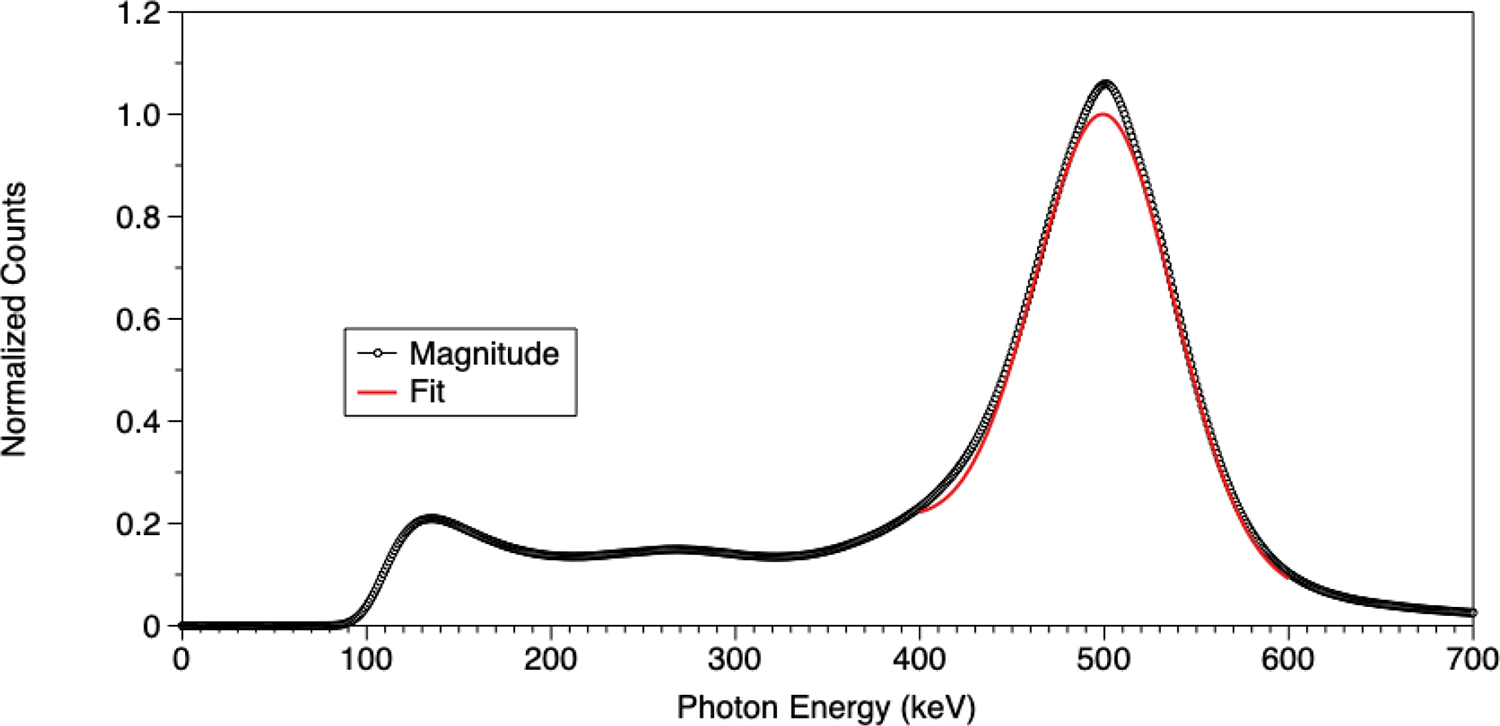
Full detector energy spectrum. Results from the fit of the photopeak to a Gaussian function is shown.

As noted above, figure 4 shows a map of detected events (*x*- and *y*-positions) acquired from the detector. Each bright peak indicates a grouping of events in a detector element. Detector efficiency is 90%. Detection uniformity is 13% across the detector’s 434.54 cm^2^ active area. Figure 5 shows a two-dimensional plot of RI. The mean RI is 0.24 ± 0.04 (range is 0.450 to 0.007). Figure 6 shows the cumulative energy spectrum for the detector. The mean energy resolution for the total detector is 18.3% FWHM. The range of energy resolution for each detector element ranges from 12.0% to 28.4% FWHM.

### 3.2 Discussion

Though the definition of a large SiPM-based detector has evolved over the last decade, the challenges to their development have remained virtually unchanged. Large area radiation detectors such as the one described in this work can be important building blocks of PET scanners by limiting overall cost and complexity with moderate effect on performance. Perhaps the most important of these challenges is related to slight variations in the gain of individual SiPM. As the number of SiPMs increases from several hundreds to thousands, to now tens of thousands, the effect of these slight differences in gain is magnified. In our design, differences in SiPM array gain are amplified by the fact that we apply the same bias voltage to a group of four arrays. To mitigate this phenomenon, we gain-matched the arrays that made up the groups, so that there were slight performance variations on an array-to-array level, as demonstrated in figure 4 and supported by the 13% detection sensitivity uniformity measured for the detector. The cooling system also contributed to the good performance. Specifically, cooling the SiPMs to ~ 16°C increases gain and reduces noise [29]. Since SiPM performance is related to device temperature, maintaining a constant temperature stabilizes these characteristics, achieving detector uniformity over time. The subtle mottled appearance of the detector element map (figure 4)) is caused by variation in the intrinsic performance of individual SiPMs in the arrays. It was not, however, possible to correct for these slight variations in the gain of individual SiPMs since we cannot control bias voltages at the individual SiPM level. Note that the grid of regularly spaced dark lines in the map shown in figure 4 coincide with the regions where individual SiPM arrays abut. These regions contain non-photosensitive plastic that is used to construct the SiPM arrays. The irregularly spaced dark lines are regions where the joining of two sub-arrays of LYSO created some optical anomalies (the large array of scintillator was constructed by combining small sub-arrays) Note that all the detector elements adjoining these regions are identifiable.

The 10% of the annihilation photons that theoretically should have been detected that were not (90% detection efficiency) were lost due to a small amount of deadtime in the detector electronics, moderate energy resolution and escape of scattered photons from the detector. The resolvability index (RI) is an indication of how well individual detector elements are distinguishable from background signal based on the detected signal, it is an alternative to the often-used peak-to-valley ratio. For example, an RI of 0.5 means that the minimum value at the mid-point between two elements is 12.5% of the maximum intensity measured for a detector element. It is equivalent to a peak-to-valley ratio of 8. Thus, the value of 0.24 ± 0.04 measured for this detector indicates a high level of the identification of individual detector elements across the surface of the device. The capability to accurately identify the location of photon interaction in the scintillator array is one of the main contributors to achieving good spatial resolution for nuclear medicine scanners.

The map shown in figure 5 illustrates the spatial distribution of RI values. The largest (>~ 0.35) RIs, indicating the lowest element resolvability, were measured mostly at the gaps where the SiPM arrays touch. This effect is most likely caused by the loss of optical photons at the junctions between two or four SiPMs, where they impinge upon regions between SiPM arrays that are not light sensitive. The lowest values (<~ 0.35), indicating the highest element resolvability, are generally located at the center of SiPM arrays where relatively few optical photons go undetected. Finally, the 18.3% FWHM mean energy resolution compares well with those reported for small detector modules. The energy resolution is slightly reduced by our use of the diode based four channel readout that clips some of the light distribution detected by the SiPMs. These results compare well with those reported for other, though smaller, SiPM-based radiation detectors designed for PET applications. For example, Belcari, et al. reported an energy resolution of 20% and RI of 0.2 for their 2.72 × 2.72 cm^2^ module [32]. Du et al. reported an energy resolution of 21.8% FWHM for a 1.55 × 1.55 cm^2^detector [33]. Li, et al. reported an energy resolution of 16.13% for their 5.38 × 5.78 cm^2^detector module [34]. Finally, Calva-Coraza, et al. constructed at an SiPM-based detector 5.74 × 5.74 cm^2^ that was reported to have an RI of 0.42 and 9.6% energy resolution [35]. The very good energy resolution is likely due to use of non-diode based SiPM readout electronics. These performance parameters compare well to those measured for our detector (energy resolution of 18.3%, RI = 0.24 ± 0.04, detection uniformity = 13% and detection efficiency = 90%). It is also important to note that these results were reported for detectors with areas ranging from 2.40 cm^2^ to 32.95 cm^2^ compared to our detector with over an order of magnitude larger sensitive area (434.54 cm^2^).

## 4 Conclusion

In this investigation we explored the challenges posed by construction of a large (32.26 × 13.47 cm^2^) panel PET detector that could be used to create a rotating or stationary ring scanner. One of these challenges is limiting, as much as possible, the amount of electronics necessary to operate the detector to reduce complexity and cost. To address this issue, we utilized a 64-to-4 multiplexed SiPM readout scheme to reduce the total number of data channels from 3,840 to 240. Additionally, data acquisition electronics were reduced by grouping the SiPM arrays in sets of four. Since this grouping resulted in a common bias voltage for the four arrays, it was important to gain match the arrays. The large amount of heat generated by the sixty SiPMs along with their associated readouts necessitated the implementation of a cooling system to maintain a constant, low operating temperature, stabilizing and enhancing SiPM performance. These efforts resulted in performance equivalent to much or exceeding much smaller PET detector modules.
